# Electrical Ear Canal Stimulation as a Therapeutic Approach for Tinnitus—A Proof of Concept Study

**DOI:** 10.3390/jcm13092663

**Published:** 2024-05-01

**Authors:** Jana Vater, Moritz Gröschel, Agnieszka J. Szczepek, Heidi Olze

**Affiliations:** Department of Otorhinolaryngology, Head and Neck Surgery, Charité-Universitätsmedizin Berlin, Corporate Member of Freie Universität and Berlin Humboldt Universität zu Berlin, Charitéplatz 1, 10117 Berlin, Germany; jana.vater@charite.de (J.V.); moritz.groeschel@charite.de (M.G.); agnes.szczepek@charite.de (A.J.S.)

**Keywords:** tinnitus, electrical stimulation, cochlea, extracochlear stimulation, anti-tinnitus implant

## Abstract

**Background:** Tinnitus—the perception of sound despite the absence of an external source—can be a debilitating condition for which there are currently no pharmacological remedies. Our proof of concept study focused on the immediate effects of non-invasive electrical stimulation through the ear canal on loudness and tinnitus-induced distress. In addition, we aimed to identify variables that may affect the simulation outcomes. **Methods:** Sixty-six patients (29 women and 37 men, mean age 54.4 ± 10.4) with chronic tinnitus were recruited to the tertiary referral hospital between December 2019 and December 2021. They underwent 10 min of electrical stimulation through the ear canal for three consecutive days. Visual analog scales measured loudness and tinnitus-induced distress immediately before and after stimulation. **Results**: After three days of electrical stimulation, tinnitus loudness decreased in 47% of patients, 45.5% reported no change, and 7.6% reported worsening. Tinnitus severity decreased in 36.4% of cases, 59.1% of patients reported no change, and 4.5% reported worsening. Women responded positively to therapy earlier than men. In addition, tinnitus distress decreased in patients with compensated tinnitus but not in those with uncompensated tinnitus. Finally, patients with bilateral tinnitus improved earlier than those with unilateral tinnitus, and the age of the patients did not influence the stimulation results. **Conclusions**: Our proof of concept study confirms the potential of non-invasive electrical stimulation of the ear as a promising screening approach to identifying patients for more advanced electrostimulation treatment, such as an extracochlear anti-tinnitus implant. These findings have practical implications for tinnitus management, offering hope for improved patient care.

## 1. Introduction

Tinnitus is the subjective perception of a sound without an external source [1] and is a symptom that can be caused by various conditions (e.g., hearing loss, cardiovascular or neurological disease, thyroid disease, diabetes, or cancer). Several etiological factors were identified for tinnitus, such as peripheral differentiation and synaptopathy, spontaneous ascending activity, GABA-ergic deficiency, or cholinergic excess, all likely contributing to central plasticity [2]. However, in clinical settings, the cause of tinnitus frequently remains unknown, in which case tinnitus is idiopathic. There is currently no drug treatment available for this condition. Sometimes, causal treatments can effectively reduce or even eliminate tinnitus. A good example is auditory therapy, such as fitting hearing aids [3,4] or auditory rehabilitation with cochlear implants [5] in patients with tinnitus caused by hearing loss.

There are currently no FDA-approved pharmacologic treatments for tinnitus. Cognitive and multimodal behavioral therapies are used to reduce the psychological distress caused by tinnitus and to prevent or treat psychological complications. The need for such treatment is determined by the level of distress caused by tinnitus and existing or developing comorbidities. However, even with all therapeutic options, patients with high tinnitus burden may experience severe limitations in daily life and work ability [6,7]. In addition to decreased quality of life, tinnitus is associated with high socioeconomic costs [8]. Therefore, a reliable and effective treatment for tinnitus would be of significant medical and socioeconomic importance.

Therapeutic approaches using electrical stimulation of the cochlea to reduce tinnitus perception have been developed since the 1970s [9,10]. In addition, numerous studies that have analyzed the effects of cochlear implants (CI) on tinnitus have found them effective in reducing tinnitus loudness [11,12,13,14]. However, despite the evidence that CI can reduce tinnitus, it is unclear whether the reduction is achieved by counteracting peripheral deafferentation [15], electrical stimulation via an implanted electrode [16], or by the interaction of both. In addition, although several clinical studies have provided data on partial or complete reduction of tinnitus in patients with cochlear implants, it is not yet clear before implantation which patients will respond positively to tinnitus reduction after CI activation. Research suggests an association between a higher degree of hearing loss and a greater likelihood of tinnitus improvement after cochlear implantation [17]. Still, this is not a rule that always works.

The literature confirms the beneficial effects of cochlear implants on tinnitus in many CI users [11,13,18]. In addition, there is evidence that auditory rehabilitation with CI improves quality of life and reduces stress and psychological comorbidities [18]. Therefore, developing an extracochlear implant for tinnitus patients, regardless of the degree of hearing loss, is essential in clinical audiology. Hence, the need emerged to create a system that could electrically stimulate the inner ear in tinnitus sufferers without the necessity for irreversible tissue damage—for example, in the form of a micro-implant placed on the round window. As part of the “INTAKT” initiative funded by the German Federal Ministry of Education and Research and dedicated to designing implants to help resolve various physiological deficits, the current project focused on determining the feasibility and effectiveness of external electrical stimulation through the ear canal in reducing tinnitus. Another project goal was to identify factors that may influence the anti-tinnitus efficacy of electrical stimulation. Obtaining such information is critical to developing an extracochlear implant for treating tinnitus and is an essential contemporary topic in clinical audiology.

To address these issues, the current proof-of-principle feasibility study aimed to determine if electrical stimulation using an electrode placed in the ear canal could decrease loudness and tinnitus-induced distress in human subjects. In addition, the results were analyzed based on the type of stimulation, the subjects’ gender, and the severity or laterality of the tinnitus.

## 2. Materials and Methods

### 2.1. Study Design

The Charité Universitätsmedizin Berlin Ethics Committee (permit number EA1/125/18 obtained on 15 January 2019) approved this prospective proof-of-principle study conducted between 2019 and 2021. The study design reflected the early investigation stage of research [19,20] and was uncontrolled. Because two different electrical stimulation schemes were used, the patients were randomly (single-blinding) assigned to each group (Stimulation Groups 1 or 2).

### 2.2. Sample Description

Inclusion criteria;

- Age 18 years or older;
- Diagnosis of chronic subjective idiopathic tinnitus;
- No physical pathologies affecting the auditory system (e.g., vestibular schwannoma, meningioma, vascular compression of the hearing nerve, vascular abnormalities in the CNS, multiple sclerosis);
- Willingness to participate and a signed informed consent;

Exclusion criteria;

- Age below 18 years;
- Diagnosis of acute idiopathic tinnitus;
- Diagnosis of objective tinnitus;
- Eardrum perforation;
- Diagnoses of cancer.

Sixty-six subjects diagnosed with chronic idiopathic tinnitus (minimum six-month duration) were included in the study based on the above inclusion-exclusion criteria. The sample characteristics are presented in Table 1. During the pre-study diagnostic process in the specialized outpatient unit, the patients underwent a detailed psychoneurological examination, including magnetic resonance imaging, to exclude possible organic causes for their tinnitus, such as schwannomas, meningiomas, or vascular malformations. The age difference between the sex groups was not statistically significant (Mood’s Median Test X^2^ = 0.696; *p* = 0.406). None of the patients had diabetes; 26 had cardiovascular and 12 had thyroid gland conditions. The patients with moderate and moderately severe hearing loss were fitted with hearing aids.

### 2.3. Electrical Stimulation Conditions

**Figure 1:**
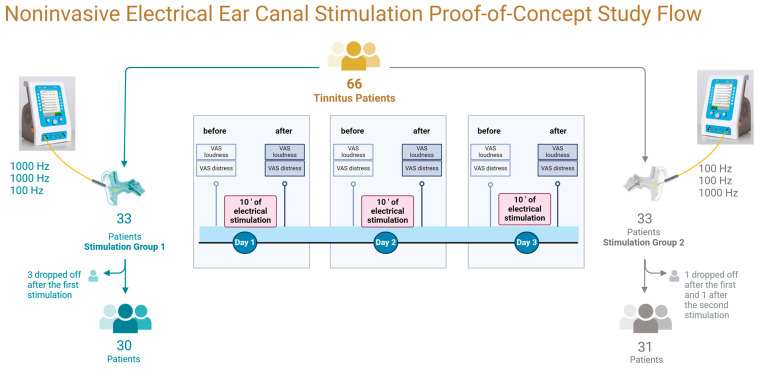
Schematic presentation of the study flow. Sixty-six tinnitus patients were randomly assigned to two stimulation groups. Both groups underwent ten minutes of electrical stimulation in the ear canal on three consecutive days. In the Stimulation Group 1, 1000 Hz was applied on two days and 100 Hz on the third day. In the Stimulation Group 2, 100 Hz was applied on two days and 1000 Hz on the third day. Immediately before and after stimulation, patients were asked to rate their subjective tinnitus loudness and tinnitus-related distress on a visual analog scale (VAS). Created with Biorender.com.

The appointments were scheduled on three consecutive days (Figure 1). They were conducted in an outpatient audiology clinic where patients underwent 10 min of stimulation with an ear canal electrode by a physician and an audiologist. In preparation for stimulation, patients were asked to lie on their backs and turn their heads to the side to comfortably insert the electrode into the ear canal. The electrode carrier constructed in the Department of ORL consisted of two flexible arms inserted into the ear canal. The arms unfolded in the ear canal and stabilized over the opposite ear canal walls. The electrode did not touch the ear canal tissues or the eardrum. Sterile saline (Freka Drainjet, cat. # 1313041, Fresenius Kabi Deutschland GmbH, Bad Homburg, Germany) was dripped into the ear canal to allow the current to flow.

Electrical stimulation was applied to one ear only in patients with unilateral tinnitus on the affected side and patients with bilateral tinnitus on the side subjectively perceived as “worse”. The stimulation gold electrode was inserted 1–1.5 cm into the ear canal and secured to the outer ear with a clip. The neutral electrode was placed on the forehead and fixed with a sterile surgical patch. During the gradual increase in current intensity, patients were asked to report possible side effects of the stimulation. These included burning or pain on the forehead where the neutral electrode was placed. In such cases, the current intensity was immediately reduced.

The C2 XTEND generator (inomed Medizintechnik GmbH, Emmendingen, Germany) randomly applied electrical stimulation with either 100 Hz or 1000 Hz frequency on the first two stimulation days (see Table 2 for details). The other frequency was used on the third stimulation day. This created two stimulation groups. Group 1 received stimulation at 1000 Hz for the first two sessions and 100 Hz for the third session. Group 2 received stimulation at 100 Hz during the first two sessions and at 1000 Hz during the third session.

The initial stimulation current intensity was 0.01 mA, gradually increasing to 3.0 mA. The average current used was 1.5 mA.

### 2.4. Assessment of Tinnitus Severity, Loudness, and Tinnitus-Induced Distress

Tinnitus severity level was assessed at admission using the Tinnitus Questionnaire (TQ). TQ was initially developed by Hallam et al. [22], and here, its validated German version was used [23]. The TQ measures the degree of tinnitus-induced distress, and its scores can range from 0 to 84. A clinically relevant system for tinnitus classification based on the total TQ score was developed, in which the TQ cutoff score of 47 is used to differentiate between habituated/compensated (below 47 points) and unhabituated/decompensated tinnitus (47 points and above). Because of technical reasons, the TQ scores were available only for 59 patients.

Patients rated tinnitus loudness and tinnitus-induced distress using the Visual Analog Scale (VAS), where 1 = very quiet, 10 = very loud, 1 = not bothersome, and 10 = very bothersome, just before and immediately after stimulation.

### 2.5. Dropouts

Five patients did not complete this study (Figure 1). Two patients from Stimulation Group 1 experienced worsening tinnitus loudness and distress immediately after the first stimulation, and one from Stimulation Group 2 after the second stimulation. They did not attend the consecutive appointments. One patient (Stimulation Group 1) with no changes after the first stimulation missed the other two appointments. One patient (Stimulation Group 2) experienced total suppression of tinnitus loudness and distress during the first stimulation and discontinued the stimulation.

### 2.6. Statistics

Statistical calculations were performed using IBM SPSS version 29.0 (IBM Deutschland GmbH, Ehningen, Germany). Because the data were not normally distributed, nonparametric statistical tests were performed.

## 3. Results

### 3.1. Electrostimulation-Induced Changes in Tinnitus Loudness and Tinnitus Distress

**Figure 2:**
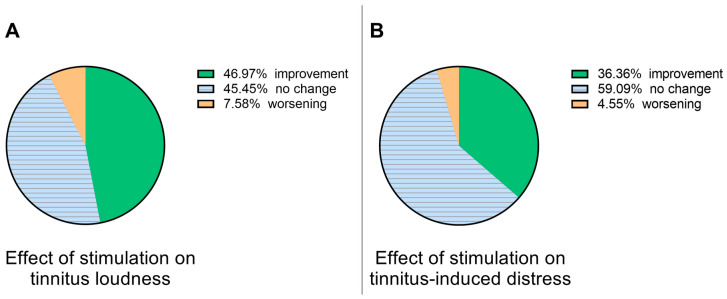
Effectiveness of electrical stimulation therapy in individual patients concerning tinnitus loudness (**A**) and tinnitus-induced distress (**B**).

We first analyzed the data regarding the benefit of electrical stimulation for individual patients. When comparing the VAS score from before the first stimulation to those reported after the third stimulation, 31 (47%) patients reported improvement, 30 (45.5%) no change, and 5 (7.6%) subjective worsening of tinnitus loudness (Figure 2A). VAS scores for tinnitus-induced distress indicated improvement in 24 (36.4%) cases, no change in 39 (59.1%), and worsening in 3 patients (4.5%) (Figure 2B).

**Figure 3:**
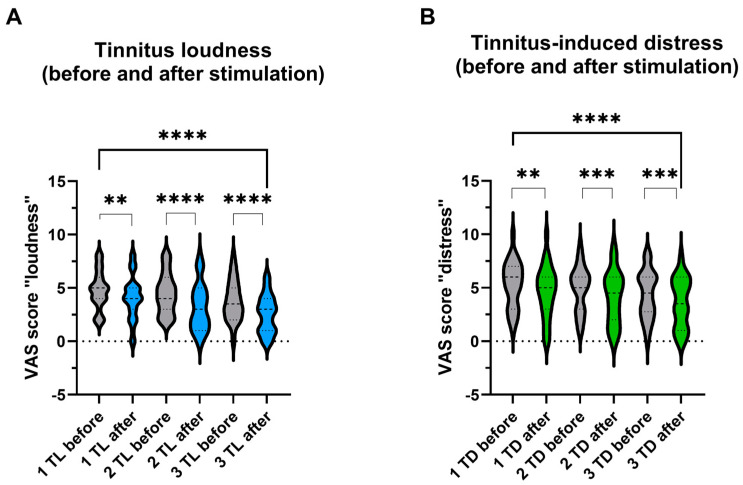
Changes in tinnitus loudness and distress after each stimulation session were registered in patients who reported improvement after stimulation (*n* = 31). Mean values of visual analog scale (VAS) scores reflecting subjective tinnitus loudness (**A**) and tinnitus-induced distress (**B**) for the entire cohort are shown as violin plots. The Wilcoxon matched-pairs signed rank test measured the significance of differences before and after stimulation; TL, tinnitus loudness, TD, tinnitus-induced distress, 1, 2, and 3 refer to the stimulation days; ** *p* < 0.05, *** *p* < 0.01, **** *p* < 0.001, ns—not significant.

There were statistically significant differences between the VAS scores before and after the stimulation for the entire cohort regarding tinnitus loudness and tinnitus-induced distress. A Wilcoxon matched-pairs signed rank test showed that the subjective tinnitus loudness decreased (Figure 3A) when measured immediately after the first stimulation (*Z* = −2.918, *p* = 0.028), after the second stimulation (*Z* = −4.424, *p* < 0.001), and after the third stimulation (*Z* = −4.192, *p* < 0.001). There was also a significant decrease in loudness measured between before the first and after the last stimulation (*Z* = −4.839, *p* < 0.001).

Similarly to tinnitus loudness, changes in the tinnitus-induced distress were significant after the first stimulation (*Z* = −2.859, *p* < 0.05) and after the second (*Z* = −3.114, *p* < 0.01) and third stimulation (*Z* = −3.241, *p* < 0.001). There was also a significant difference in tinnitus-induced distress measured between the first and after the last stimulation (*Z* = −4.086, *p* < 0.001) (Figure 3B).

### 3.2. Lack of Difference in Outcome between the Stimulation Groups

**Figure 4:**
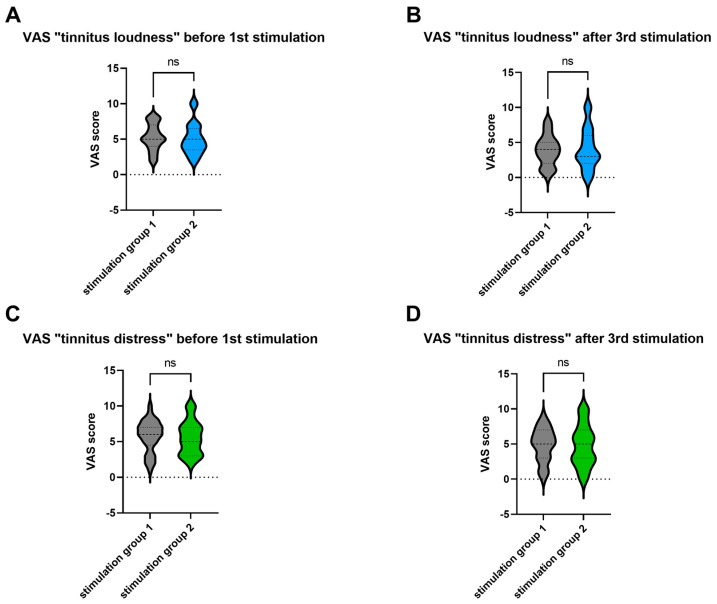
Visual analog scale (VAS) scores indicating tinnitus loudness or distress were compared between stimulation groups 1 and 2 (n = 66). The VAS scores indicating “tinnitus loudness” were measured immediately before the first stimulation (**A**) or immediately after the third stimulation (**B**). The VAS scores indicating “tinnitus distress” were measured immediately before the first stimulation (**C**) or immediately after the third stimulation (**D**). The scores are shown separately for each stimulation group. Using the Mann–Whitney U test, no significant differences were found between the groups, either at baseline (Figure 3A,C) or at the end of the study (Figure 3B,D). ns—not significant.

The results of the two stimulation groups were compared to determine whether the sequence of stimulation frequencies (100 or 1000 Hz) influences the loudness or distress of tinnitus. The comparison of the VAS before and after the third stimulation showed a statistically significant improvement for both measured parameters and no significant differences between the groups. A Mann–Whitney U test was performed to evaluate whether VAS scores for tinnitus loudness and distress differed by stimulation group (Figure 4). The results indicated no significant difference between the VAS scores before or after stimulation in stimulation group 1 and stimulation group 2. These results suggest that the order of electrical stimulation (first 100 Hz and then 1000 Hz, or vice versa) is irrelevant to the outcome.

### 3.3. Positive Response to Electrical Stimulation Is Observed Earlier in Women than in Men

The comparison between the VAS before the first stimulation and the VAS after the third stimulation with a sex split showed a statistically significant improvement in both measured parameters for both sexes.

The difference in VAS scores between the gender groups concerning the beginning of the study (VAS tinnitus loudness Mood’s Median Test X^2^ = 0.679; *p* = 0.410; VAS tinnitus distress Mood’s Median Test X^2^ = 0.097; *p* = 0.905) or study endpoint (VAS Tinnitus Loudness Mood’s Median Test X^2^ = 0.295; *p* = 0.587; VAS Tinnitus Distress Mood’s Median Test X^2^ = 0.208; *p* = 0.648) was not statistically significant.

There was a difference in the timing of response to electrical stimulation between the sexes. Women reported reduced tinnitus loudness immediately after the first ear stimulation (and after subsequent sessions), whereas men responded positively only after the second and third electrical stimulations (Table 3).

### 3.4. Stimulation Results Vary between Groups with Different Levels of Tinnitus Severity

The breakdown of subjects by TQ score showed 37 patients with compensated (habituated) tinnitus (56.1%) and 22 patients with decompensated (unhabituated) tinnitus (33.3%). No TQ score was available for seven patients (10.6%). The Wilcoxon-matched pairs signed ranks test with stratification based on compensation/habituation criteria showed differences in the outcome of electrical stimulation between the groups. Patients with compensated (habituated) tinnitus benefited from stimulation therapy, as measured by both VAS domains (tinnitus loudness and tinnitus-induced annoyance). Patients with decompensated tinnitus benefited from the treatment concerning tinnitus loudness but not tinnitus-induced distress (Table 4).

### 3.5. Stimulation Results Vary between Patients with Unilateral and Bilateral Tinnitus

When the sample was analyzed according to the laterality of the tinnitus, no difference was found between the groups’ median TQ or VAS scores (before the first stimulation). However, there was a significant difference in stimulation outcome. The Wilcoxon matched-pairs signed rank test showed that in the unilateral tinnitus group, a significant reduction in loudness was observed only after the third stimulation (*Z* = −2.121, *p* = 0.034). In contrast, in the bilateral tinnitus group, a significant improvement was observed after the first (*Z* = −2.498, *p* = 0.012) as well as after the second (*Z* = −4.283, *p* < 0.001) and third stimulation (*Z* = −3.992, *p* < 0.001). We found a significant overall improvement in both groups when comparing the VAS scores before the first stimulation with those after the third stimulation.

Similar results were obtained regarding tinnitus-induced distress. The Wilcoxon matched-pairs signed rank test indicated significant improvement in patients with bilateral tinnitus after the first (*Z* = −2.088, *p* = 0.037), second (*Z* = −3.114, *p* = 0.002), and third (*Z* = −2.971, *p* < 0.001) stimulation. Patients with unilateral tinnitus improved significantly after the third stimulation (*Z* = −2.000, *p* = 0.046). Only the group with bilateral tinnitus significantly improved when comparing the VAS scores before the first and after the third stimulation.

### 3.6. Age, Duration of Tinnitus and the Degree of the Hearing Loss Do Not Affect the Stimulation Outcome

We calculated Spearman’s rank-order correlation to determine if the stimulation outcome (effect on tinnitus loudness and tinnitus-induced distress) is influenced by the patient’s age, duration of tinnitus, or hearing loss. None of the variables tested (age, tinnitus duration, grade of hearing loss, or TQ score at admission) influenced the outcome (Table 5). The grade of hearing loss was correlated with the patient’s age and TQ score at admission.

## 4. Discussion

In the current proof-of-concept study, we wanted to determine if non-invasive electrical stimulation through the ear canal can suppress loudness and tinnitus-induced distress. Moreover, we sought to determine the factors influencing the suppressing effect. The study was designed to deliver proof of concept and demonstrate the feasibility of electrical stimulation in reducing tinnitus loudness and distress. The factors tested included the frequency of current used for stimulation, the sequence of application of different stimulation currents, tinnitus grade at admission, tinnitus laterality, sex, and age of the patients. Some intermediate results obtained during this project have already been reported in the literature [20] to satisfy the funding agency’s requirements.

Analysis of the electrical stimulation results showed that 47% of patients in our study experienced a statistically significant reduction in tinnitus loudness. Other studies using a similar electrical stimulation system to reduce tinnitus have had mixed results. Mielczarek et al. performed two studies. The first included six patients who received unilateral stimulation, after which improvement was noted in 83.3% of the ears and no change in 16.7% [24]. In the second study, the ears of 28 patients with tinnitus were tested individually, and a reduction in tinnitus intensity was found in 75%, no change in 18%, and worsening in 7% [25]. The study by Zeng et al. [26] included ten patients with tinnitus, four of whom received non-invasive stimulation via the outer ear. Two patients reported no change during or after stimulation. The other two reported a slight reduction in tinnitus during stimulation, and one found its complete disappearance after stimulation. Finally, a study by Suh et al. [27] in 14 tinnitus patients showed an average 22% reduction in tinnitus when stimulated through the ear canal. There are fundamental differences between our current study and those conducted by other researchers. The first difference is in the number of patients, which were 66 in our study, 6 and 28 in Mielczarek’s study, 4 in Zeng’s study, and 14 in Suh’s study. Not only the number but also the age of the patients varied (mean 54.4 ± 10.44 years in our study, 53.4 ± 15.6 and 58.5 ± 11.83 years in Mielczarek’s study, 61.5 ± 9.25 years in Zeng’s study, and 44 years in Suh’s study with no SD mentioned).

Other differences are the parameters used for stimulation, such as frequency (100 or 1000 Hz for us, 250 Hz [24], or 0.25, 1, 2, 3, 4, 5, 6, 7, and 8 kHz [25] for Mielczarek, 10 to 10,000 Hz for Zeng and 0.01 to 10 kHz for Suh), as well as stimulation duration (10 min for us, not specified for Mielczarek, 2–3 min and sometimes longer for Zeng, and 3 min or longer for Suh). In addition, due to a lack of suitable commercially available equipment, we and other groups used homemade stimulation devices, which could have also contributed to differences in the results.

The frequency of the current used (100 Hz or 1000 Hz) and the sequence of its application did not produce significant differences in reducing tinnitus loudness or distress under the applied conditions. The subjective loudness of the tinnitus measured in the entire group had already decreased significantly on the first day after the 10-min stimulation and on the second and third days. In contrast, tinnitus distress did not decrease significantly until the second and third days of stimulation.

Women reported reduced tinnitus loudness after the first, second, and third stimulation. In contrast, men reported a reduction after the second and third stimulation. Gender differences in sensory reactivity to external electrical stimulation are known to explain the greater sensitivity to pain in women [28,29], likely due to differences in body fat or water content [30]. This type of study has not yet been conducted in audiological research. Still, the physical factors affecting women’s greater sensitivity to electrical stimulation could likely be universal, explaining the results of our study.

We also found that patients with compensated/habituated and decompensated/unhabituated tinnitus reported significantly reduced loudness after the second and third stimulations. Despite this, only patients with compensated/habituated tinnitus experienced a significant reduction in tinnitus distress after the 3 days of electrical stimulation. No comparable data are available in published research; however, it is known that the emotional status of patients affects both loudness and tinnitus-induced distress [31,32] and can be subject to fluctuations [33]. We hypothesize that the patients with decompensated/unhabituated tinnitus may have negative valence and emotional status that contribute to tinnitus-induced distress but are not a target of electrical stimulation. However, since the emotional status of the patients was not the subject of our study, we cannot confirm or reject such a hypothesis.

There were differences in stimulation efficacy between patients with unilateral and bilateral tinnitus. The latter group reported a reduction in tinnitus loudness after the first stimulation as well as the second and third stimulations. In contrast, patients with unilateral tinnitus improved only after the third stimulation. We thought this result could be attributed to possible differences in tinnitus habituation between the groups. Still, the proportion of compensated/habituated cases within the two groups (7 cases (58.3%) in the unilateral group and 30 (55.6%) in the bilateral group) were comparable and cannot explain the effect seen. However, our sample was relatively small, and no data were available on compensation for three patients with unilateral tinnitus (25% of the group) and four patients in a bilateral group (7.4%). Different results were obtained by Genitsaridi et al. [34] and Song et al. [35], who suggested that unilateral tinnitus is more distressing than bilateral. Nevertheless, our observations are consistent with those made by Aazh et al. [36], who used a large sample of patients (n = 311) with unilateral and bilateral tinnitus for the comparative analysis and did not find differences in tinnitus severity between the two groups by Tinnitus Handicap Inventory THI. Furthermore, sex distribution was similar within the groups (W/M 33%/67% in unilateral and 46%/54% in bilateral group), therefore unlikely accounting for the effect seen. We also sought to explain the differences in the efficacy of electrical stimulation on tinnitus-induced distress in terms of audiological parameters. Using degrees of hearing loss in the comparative analysis, we found no explanation for the observed differences, which agrees with the study of Yang et al. [37]. Aazh et al. pointed out the importance of interaural asymmetry in unilateral but not bilateral tinnitus patients [36]. That aspect should be studied in the future in electrical stimulation. Moreover, neuropsychological differences between the patients with unilateral and bilateral tinnitus suggest significant deficits in the auditory memory and attention of the unilateral and not bilateral group [34], highlighting the disparity between unilateral and bilateral tinnitus.

Spearman’s rank-order correlation confirmed the long-known association between hearing loss and tinnitus grade [38] or age and degree of hearing loss [39]. Still, it did not reveal any association between the electrical stimulation results and the patient’s age, duration of tinnitus, degree of tinnitus, or hearing loss, suggesting complex mechanisms underlying the efficacy of stimulation.

The information gathered during this study fulfilled the expectations of the INTAKT project by providing parameters relevant to extracochlear electrical stimulation for tinnitus. This knowledge will be helpful in the future development of a tinnitus implant. In addition, the results should help select patients for cochlear implantation and may help predict tinnitus reduction after CI.

Our study is not without limitations. The first is the relatively small sample size, which should be increased. Initially, at least 100 patients were supposed to be included. However, because we conducted the study during the pandemic period (from the end of 2019 to the end of 2021), we were limited by patient access to the hospital, national and internal regulations governing the clinical research activities of tertiary healthcare centers during the pandemic, as well as the high incidence of COVID-19 among staff. The second limitation is the lack of a control group, which agrees with the proof-of-concept design but leaves questions about the placebo effect open. Future randomized controlled trials should include control, sham-stimulated tinnitus patients, who would be informed that the stimulation begins when, in fact, the equipment would be switched off. A final pitfall of our research is the insufficient audiometric information on matched tinnitus loudness and frequency and possible comorbid psychological conditions. It is recommended that such information be collected in future studies.

We envision a twofold future for this project. First, continuing the stimulation scheme described here would be essential to determine if stimulation on four or more consecutive days could extinguish or decrease the tinnitus sound and distress, as it did in one patient in our sample. It would also be essential to know how long the positive effect of electrical stimulation lasts. Second, this project was designed as a pilot one in preparation for the design of an extracochlear anti-tinnitus implant. The information gained during this study should help draw a blueprint for such a device in the near future.

## 5. Conclusions

The results of electrical stimulation through the ear canal in tinnitus patients presented in the current paper demonstrate the feasibility of such a procedure in a hospital setting and confirm the results of a few studies by other groups showing that almost half of the patients benefit from this type of therapy. The novel information provided by our research is that women respond more quickly to electrical stimulation than men, that the severity of tinnitus negatively affects the outcome of stimulation in terms of tinnitus distress but not in terms of loudness, and that the laterality of the tinnitus also contributes to the final outcome. In conclusion, non-invasive electrical stimulation through the ear canal seems to be a promising treatment for tinnitus. The results of our study suggest that further development of this type of therapy towards extracochlear tinnitus implants is warranted, as it may become a valuable therapeutic approach. Despite the benefits of the electrical stimulation provided by intracochlear stimulation with a CI, many tinnitus patients do not qualify for such surgery because they have no or only mild hearing loss. An extracochlear implant would be extremely valuable for these patients by directly eliminating the perception of tinnitus and leaving their hearing abilities intact.

## Figures and Tables

**Table 1:** Sample characteristics.

sex	women	*n* = 29	43.9%
	men	*n* = 37	45.1%
age	mean 54.4 years (SD ± 10.4)	(min 26–max 80 years)	
hearing loss	normal hearing (better than 20 dB *)	*n* = 10	15.2%
	mild (20–34 dB)	*n* = 43	65.2%
	moderate (35–49 dB)	*n* = 6	9.1%
	moderately severe (50–64 dB)	*n* = 7	10.6%
tinnitus laterality	unilateral	*n* = 12	18.2%
	bilateral	*n* = 54	81.8%
Duration of tinnitus in months (*n* = 32)	mean 28.13 (SD ± 19.18)	(min 6–max 48 or more)	
Tinnitus Questionnaire score at admission (*n* = 59)	mean 41.59 (SD ± 17.56)	(min 11–max 81)	
cardiovascular disease		*n* = 26	39.4%
thyroid disease		*n* = 12	18.2%

SD, standard deviation; *n*, number of patients; *, grading according to the updated WHO definition [21], the values consider the pure tone average in the better ear for 500, 1000, 2000, and 4000 Hz.

**Table 2:** The parameters used for each of the stimulation programs.

	100 Hz	1000 Hz
Mode	continuous	continuous
Modulation frequency	1 Hz	5 Hz
Polarity	biphasic 50	biphasic 50
Puls width	1000 µs	400 µs
Interstimulus interval	10 ms	1 ms
Carrier frequency	100 Hz	1000 Hz
Initial current value	0.01 mA	0.01 mA
Maximal current value	3.0 mA	3.0 mA

**Table 3:** The significance of changes in tinnitus loudness measured with VAS after the first, second, and third stimulation, as well as before the first and after the third simulation, was calculated using the Wilcoxon test. Two-sided asymptotic significance (*p*-value) indicated that women rated the tinnitus loudness after the first stimulation as significantly decreasing, whereas men did not.

	Men		Women	
	*Z*	*p*	*Z*	*p*
Tinnitus loudness first stimulation	−0.681	0.496	−2.121	0.034
Tinnitus loudness second stimulation	−3.211	0.001	−3.218	0.001
Tinnitus loudness, third stimulation	−3.176	0.001	−3.220	0.001
Tinnitus loudness delta first—third stimulation	−3.071	0.002	−3.657	0.001

**Table 4:** The significance of changes in tinnitus loudness and tinnitus-induced distress after the first, second, and third stimulations, as well as before the first and third simulations, was calculated using the Wilcoxon test. The sample was split based on tinnitus compensation (habituation). Two-sided asymptotic significance (*p*-value) indicated that both groups rated the loudness of tinnitus as significantly decreasing after the second and third stimulations. Only the compensated (habituated) group rated the tinnitus-induced distress as significantly decreasing after the second and third stimulations.

	Compensated (Habituated) Group		Decompensated (Unhabituated) Group	
	*Z*	*p*	*Z*	*p*
Tinnitus loudness first stimulation	−1.342	0.180	−1.065	0.287
Tinnitus loudness second stimulation	−3.286	0.001	−2.695	0.007
Tinnitus loudness, third stimulation	−3.256	0.001	−2.699	0.007
Tinnitus loudness delta first—third stimulation	−3.781	0.001	−2.590	0.010
Tinnitus-induced distress first stimulation	−1.455	0.146	−0.447	0.655
Tinnitus-induced distress second stimulation	−2.585	0.010	−1.897	0.058
Tinnitus-induced distress third stimulation	−2.716	0.007	−1.841	0.066
Tinnitus-induced distress delta first—third stimulation	−3.400	0.001	−1.781	0.075

**Table 5:** Spearman’s rank-order correlation was run to determine the relationship between tinnitus duration, patient’s age, TQ score, or the grade of the hearing loss, and the effectiveness of electrical stimulation on tinnitus loudness or tinnitus-induced distress. The significant correlations are shown in bold and identified with an asterisk.

		Tinnitus Duration in Months	Effectiveness Regarding Tinnitus Loudness	Effectiveness Regarding Tinnitus-Induced Distress	Age	TQ Score at Admission	Grade of Hearing Loss
Tinnitus duration in months	Correlation Coefficient	--					
	Sig. (2-tailed)						
	*n*	32					
Effectiveness regarding tinnitus loudness	Correlation Coefficient	0.212	--				
	Sig. (2-tailed)	0.245					
	*n*	32	66				
Effectiveness regarding tinnitus-induced distress	Correlation Coefficient	−0.142	**0** .662 *	--			
	Sig. (2-tailed)	0.438	**0** **.000**				
	*n*	32	66	66			
Age	Correlation Coefficient	0.201	0.135	0.033	--		
	Sig. (2-tailed)	0.270	0.280	0.795			
	*n*	32	66	66	66		
TQ score at admission	Correlation Coefficient	0.196	−0.012	−0.106	0.245	--	
	Sig. (2-tailed)	0.282	0.929	0.426	0.062		
	*n*	32	59	59	59	59	
Grade of hearing loss	Correlation Coefficient	0.113	−0.108	−0.130	**0** .383 *	**0** **.42** 5 *	--
	Sig. (2-tailed)	0.538	0.389	0.298	**0** **.002**	**0** **.** **001**	
	*n*	32	66	66	66	59	66

## Data Availability

The data presented in this study are available on request from the corresponding author due to ethical reasons.

## Abbreviations

CI	cochlear implant
CNS	central nervous system
dB	decibel
FDA	Federal Drug Administration
Hz	Hertz
*p*	probability
SD	standard deviation
TQ	Tinnitus Questionnaire
VAS	visual analog scale
WHO	World Health Organization
Z	a difference in the median pre- vs. post-test rank score
